# How similar are species names and why does this matter for biodiversity data

**DOI:** 10.3897/BDJ.14.e196932

**Published:** 2026-07-03

**Authors:** André Menegotto, Cristina Ronquillo, Joaquín Hortal, Thomas J. Webb

**Affiliations:** 1 Ecology and Evolutionary Biology, School of Biosciences, University of Sheffield, Sheffield, United Kingdom Ecology and Evolutionary Biology, School of Biosciences, University of Sheffield Sheffield United Kingdom; 2 Department of Biogeography and Global Change, Museo Nacional de Ciencias Naturales (MNCN-CSIC), Madrid, Spain Department of Biogeography and Global Change, Museo Nacional de Ciencias Naturales (MNCN-CSIC) Madrid Spain

**Keywords:** Damerau-Levenshtein distance, epithet, fuzzy match, scientific name, taxonomic harmonisation

## Abstract

Standardising taxonomic names is an essential step in biodiversity studies to ensure robust data aggregation under the most recent accepted species nomenclature. Fuzzy (inexact) matching is widely used in this process to detect correspondences between scientific names that differ due to alternative spelling or orthographic mistakes. Such an approach assumes that species names are sufficiently distinct such that names differing in just a few characters in fact refer to the same taxon, but this has rarely been evaluated. Across c. 230,000 marine species names, we show that name similarity is common: 19.34% of specific epithets differ by three or fewer edits from another epithet within the same genus. Shared epithets are also widespread within and across phyla, occurring in 73% of all marine species; in 6.05% of these cases, the associated genera differ by three or fewer edits. This level of similarity increases the risk of incorrect matches, limiting the reliability of automated text-string tools in biodiversity big data analyses and highlighting the importance of combining post-matching filters with systematic and authorship information in taxonomic workflows to support name resolution beyond orthographic similarity.

## Main

Due to the dynamic nature of species names, taxonomic standardisation has become a crucial step in ecological studies that utilize aggregated datasets. During this process, synonyms are consolidated under a single accepted name, while records not found in authoritative taxonomic references or considered invalid are removed, thereby ensuring the quality of biological records (Seah 2023, Sandall et al. 2023). Within this framework, homonyms represent a well-recognized challenge (identical names applied to different species can lead to the incorrect integration of biological data), often mitigated by incorporating information on higher taxa or name authority into the standardisation process (Boyle et al. 2013). However, misassignments may also arise from high similarity among distinct scientific names. Because scientific names may have alternative spellings or contain orthographic errors introduced in the original biodiversity records or during the digitization process (Rees 2014), fuzzy matching is typically used to identify corresponding names despite such variation. The problem with fuzzy matching is that when two species have similar names, the difference may be mistakenly interpreted as an orthographic error, thus creating the same kind of confusion that occurs with homonymy (i.e. the records of different species will be wrongly merged under a single name) (Grenié et al. 2023). The question that inevitably arises, therefore, is: how does name similarity affect the detection of spelling variations during standardisation procedures?

**Figure 1.**
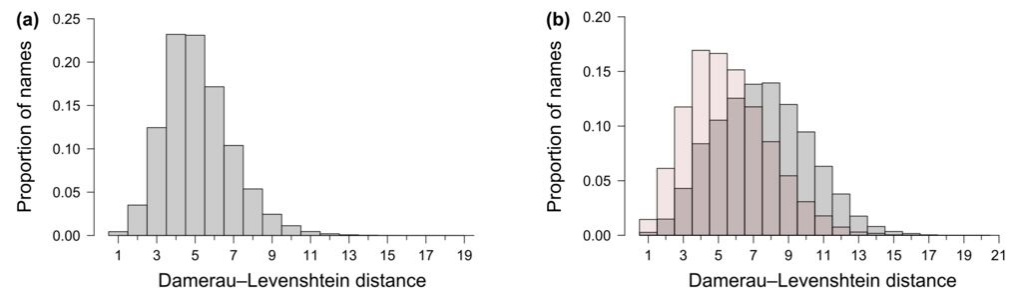
Similarity among scientific names. **a** Histogram showing the proportion of accepted species names in WoRMS (n = 229,508) with their minimal Damerau-Levenshtein distances (number of edits) to other accepted names, considering the entire string (i.e. genus + epithet). **b** The same analysis restricted to genera when the epithet is shared (grey) and to epithets when the genus is shared (red). The results show that many valid names are closely similar to others, with similarity increasing as the number of allowed edits grows. This is particularly pronounced among epithets within the same genus, compared with genera sharing the same epithet.

Using the World Register of Marine Species (WoRMS, WoRMS Editorial Board 2025), we explored this question by computing the Damerau-Levenshtein distance (Damerau 1964, Levenshtein 1966, Loo 2014) across nearly 230,000 unique and accepted binomials from 85 different phyla. Specifically, we quantified the minimum number of edits between each name and its closest orthographic match to assess how naturally similar the valid scientific names can be. Because a good fuzzy matching approach should maximize precision, we also tested how non-phonetic post-matching filters could reduce false-positive matches (see Rees 2014 for detailed description). We found that 1,026 names (0.45%) are within one edit of another accepted species name, 8,063 (3.51%) are within two edits, and more than ten percent (n = 28,564; 12.45%) are within three edits. The peak of name similarity occurs at four to five edits. After that, the number of closely matching names begins to decline (Fig. 1a). The proportion of names with multiple matches also increases with minimum edit distance, from 4% for those within one edit to 11.92%, 28.13%, and 42.43% for those within two, three, and four edits, respectively. Post-matching filters had no effect on names differing by a single edit but progressively reduced false-positive matches from two edits onward. Specifically, all 1,026 names at one edit (100% of initial search) were retained after filtering, whereas the number of false matches declined to 4,070 at two edits (50.48%), 9,060 at three edits (31.72%), and 2,622 at four edits (4.92%).

Because edits can be distributed across different parts of the name, we quantified how often names within three edits of another valid species had all edits concentrated in a single name component. Surprisingly, in 99.20% of the cases, edits were concentrated in only one component, indicating that the closest matches tend to share either the same genus with all edits in the species epithet (81.17% of cases), or they shared the same species epithet with all edits in the genus name (18.03%). The same result persisted after applying post-matching filters, with edits concentrated in a single component in 98.57% of cases, either in the epithet (77.41%) or in the genus (21.16%). This pattern suggests that most potential misassignments are likely to occur among closely related taxa rather than between distantly related organisms.

The high frequency of similar epithets within the same genus could be explained by the untested assumption that, due to the structure of the taxonomic hierarchy, species are more likely to share a genus than an epithet, thereby increasing the likelihood of spelling similarity in the second component of the binomial name rather than in the first. To explore this hypothesis, we searched for names sharing one identical component and measured the similarity in the non-shared component, i.e. the minimum distance in the epithet among species sharing the same genus, and the minimum distance in the genus among species sharing the same epithet. We found that epithets are repeatedly used to name different species (n = 24,655 from 85,863 unique epithets; total species sharing epithets = 168,300), in numbers broadly comparable to those observed for genera (n = 20,846 from 33,926 unique genera; total species sharing genus = 216,428). This indicates that similar adjectives and geographical descriptors have been widely used to name species across very different taxa (De Grave et al. 2025). For instance, the epithets 'gracilis', 'australis', 'elegans', 'japonica', and 'pacifica' have been applied to more than 400 different species each, with Arthropoda, Mollusca and Chordata containing the highest number of species with non-exclusive epithets (Suppl. material 1). Despite the large number of epithets shared across species, our results confirm that repeated genera involve more species overall and show that epithets tend to be more similar among congeneric species than genera are among species sharing the same epithet (Fig. 1b). Specifically, a similar genus occurred within shared epithets in 0.27% (≤ 1 edit), 1.76% (≤ 2 edits), and 6.05% (≤ 3 edits) of the cases, whereas similar epithets occurred within shared genera in 1.45% (≤ 1edit), 7.58% (≤ 2 edits), and 19.34% (≤ 3 edits) of the cases. The higher frequency of similar names in the epithet could indicate that sister species share similar characteristics, or it may simply reflect chance, since genera typically contain more species, increasing the probability of finding names with smaller edit distances. Regardless of the reason, these results demonstrate that allowing up to three edits within the same component greatly increases the risk of incorrectly combining two accepted species, even when non-phonetic post-matching filters are applied.

Fuzzy matching is a key component of taxonomic harmonisation, as it effectively improves name resolution and, consequently, minimises information loss (Santovito et al. 2026). In many published tools offering this functionality, misspelled names or legitimate name variants are identified using edit-distance metrics, with user-defined limits (Kindt 2020, Zhang and Qian 2023, Andrade 2026). Here, we show that increasing these limits substantially raises the likelihood of false-positive matches. To mitigate this issue, major platforms (e.g. Taxonomic Name Resolution Service, Boyle et al. 2021; WoRMS, WoRMS Editorial Board 2025) usually apply post-matching filters as implemented in the Taxamatch algorithm (Rees 2014). These filters markedly reduce the number of erroneous matches, but our results indicate that such mismatches are not completely eliminated. Together, these findings reinforce the need to incorporate two additional sources of information when standardising biodiversity records. One is the systematic background, which can be used to account for systematic correspondences or to restrict searches to names within a given taxonomic group. However, the high frequency of similar names within genera highlights that including authorship is also essential to avoid mistaking close-related species for typographical variants.

## Data availability

The primary data used in this study is available at the WoRMS website (https://www.marinespecies.org/). The code and data underpinning the analyses reported in this paper are deposited in the Zenodo repository at https://doi.org/10.5281/zenodo.20796133.

## Supplementary Material

39E06030-C0DC-5681-A3E3-301138BD9692 10.3897/BDJ.14.e196932.suppl1 Supplementary material 1 Distribution of epithets shared among different species Data type Figure Brief description Panel with four plots showing the frequency of epithets shared among species, the most common shared epithets, and the phyla in which shared epithets are most frequent. File: oo_1601224.pdf https://binary.pensoft.net/file/1601224 Menegotto A., Ronquillo C., Hortal J., Webb T.J.
