# Supplementary material for: How similar are species names and why does this matter for biodiversity data
Source: Biodivers Data J. 2026 Jul 3;14:e196932. doi: 10.3897/BDJ.14.e196932 (PMC13354977; doi:10.3897/BDJ.14.e196932)
Supplement: Supplementary material 1 — Distribution of epithets shared among different species [file bdj-14-e196932-s001.pdf]

Supplementary Material

How similar are species names and why does this matter for biodiversity data

Menegotto A., Ronquillo C., Hortal J., Webb T.J.

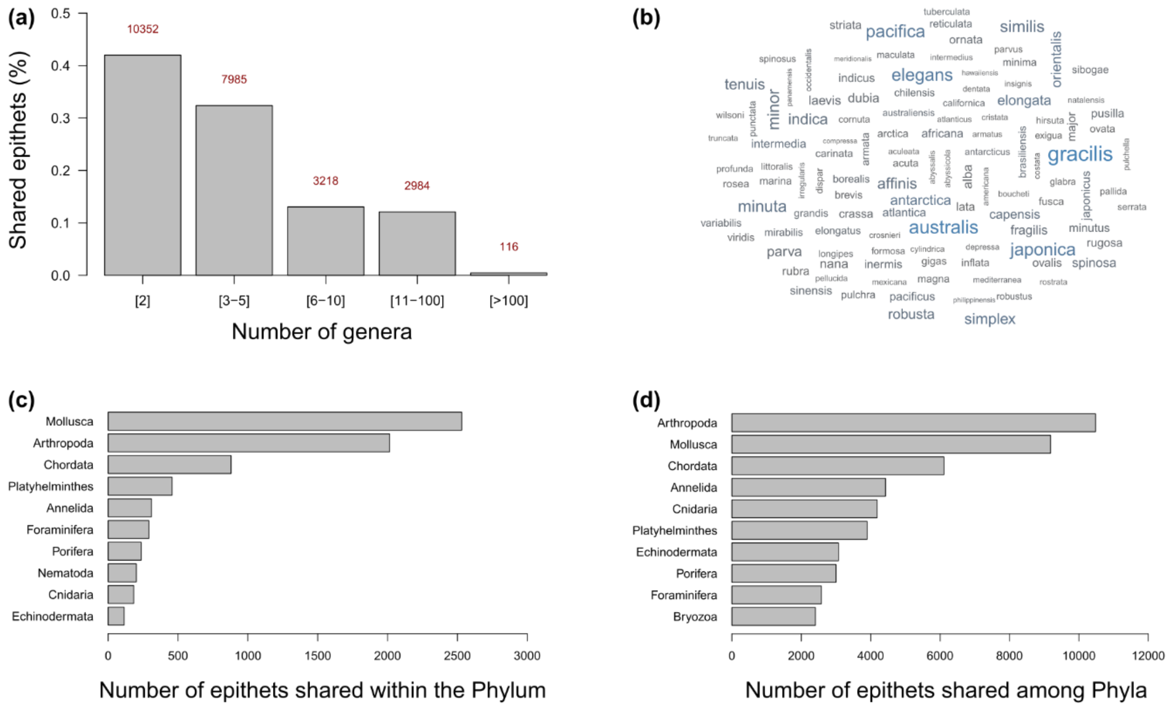

Figure S1. Distribution of specific epithets shared among different species. (a) Bar plot showing the number of genera in which shared epithets occur. The y-axis represents the percentage of epithets among those occurring in more than one genus (n = 24,655), and red labels above the bars indicate raw counts. (b) Word cloud of epithets occurring in more than 100 species. Epithets shown in blue and with larger font sizes occur more frequently. (c) Number of epithets occurring more than once across different species but restrict to a single phylum. (d) Number of epithets occurring more than once across different species and shared among different phyla. For panels (c) and (d), only the 10 phyla with the highest number of cases are shown.
